# Supplementary material for: Deletion of wfs1 Impairs Oligodendrocyte Precursor Cells Dorsal Distribution and Myelination Through the wfs1-hmgcs1 Axis in Zebrafish
Source: Biology (Basel). 2026 Aug 16;15(16):1402. doi: 10.3390/biology15161402 (PMC13510151; doi:10.3390/biology15161402)
Supplement: Supplementary file 1 [file biology-15-01402-s001.zip › File S1-Transcriptome sequencing data and differentially expressed genes (DEGs) in wfs1-- zebrafish at 5 dpf/1.GO/wfs1KOvsWT/GSEA/WT/MACROAUTOPHAGY/MACROAUTOPHAGY(GO_0016236).html]

Details for gene set MACROAUTOPHAGY(GO:0016236)[GSEA]


|  || Dataset | deg.sample |
| Phenotype | sample.cls |
| Upregulated in class | WT |
| GeneSet | MACROAUTOPHAGY(GO:0016236) |
| Enrichment Score (ES) | -0.38881156 |
| Normalized Enrichment Score (NES) | -1.7384194 |
| Nominal p-value | 0.0 |
| FDR q-value | 0.36699516 |
| FWER p-Value | 0.782 |
Table: GSEA Results Summary

  

Fig 1: Enrichment plot: MACROAUTOPHAGY(GO:0016236)
  
Profile of the Running ES Score & Positions of GeneSet Members on the Rank Ordered List

  

| PROBE | DESCRIPTION   (from dataset) | GENE SYMBOL | GENE\_TITLE | RANK IN GENE LIST | RANK METRIC SCORE | RUNNING ES | CORE ENRICHMENT || 1 | 415219 | rab1bb |  |  | 516 | 2.933 | 0.0399 | No |
| 2 | 555795 | si:ch211-260e23.9 |  |  | 1960 | 1.384 | 0.0209 | No |
| 3 | 569810 | smg1 |  |  | 2490 | 1.177 | 0.0268 | No |
| 4 | 321965 | taz |  |  | 3242 | 0.984 | 0.0220 | No |
| 5 | 492808 | stx17 |  |  | 3434 | 0.945 | 0.0341 | No |
| 6 | 101243556 | tmem173 |  |  | 3847 | 0.860 | 0.0376 | No |
| 7 | 566914 | rab1aa |  |  | 4535 | 0.739 | 0.0301 | No |
| 8 | 100330105 | map1lc3cl |  |  | 5232 | 0.652 | 0.0206 | No |
| 9 | 541406 | lamp2 |  |  | 7906 | 0.431 | -0.0552 | No |
| 10 | 791223 | atg9b |  |  | 8583 | 0.396 | -0.0689 | No |
| 11 | 324254 | mtor |  |  | 9164 | 0.355 | -0.0803 | No |
| 12 | 100329702 | ulk2 |  |  | 9563 | 0.329 | -0.0866 | No |
| 13 | 406466 | map1lc3a |  |  | 9731 | 0.319 | -0.0857 | No |
| 14 | 751627 | rab43 |  |  | 9800 | 0.316 | -0.0818 | No |
| 15 | 394117 | rab1ba |  |  | 9810 | 0.315 | -0.0761 | No |
| 16 | 568021 | tp53inp1 |  |  | 10439 | 0.276 | -0.0905 | No |
| 17 | 393578 | ufl1 |  |  | 11203 | 0.233 | -0.1101 | No |
| 18 | 100537478 | ubxn2a |  |  | 11421 | 0.221 | -0.1127 | No |
| 19 | 100000277 | atg2b |  |  | 11711 | 0.205 | -0.1178 | No |
| 20 | 100332174 | rb1cc1 |  |  | 12241 | 0.177 | -0.1311 | No |
| 21 | 558010 | lix1 |  |  | 12304 | 0.174 | -0.1297 | No |
| 22 | 336632 | atg3 |  |  | 12708 | 0.154 | -0.1395 | No |
| 23 | 337220 | emc6 |  |  | 12859 | 0.147 | -0.1414 | No |
| 24 | 559391 | nprl2 |  |  | 13113 | 0.135 | -0.1467 | No |
| 25 | 555970 | snx14 |  |  | 13277 | 0.126 | -0.1495 | No |
| 26 | 100004597 | phf23b |  |  | 13280 | 0.126 | -0.1471 | No |
| 27 | 100329883 | fyco1b |  |  | 13392 | 0.121 | -0.1483 | No |
| 28 | 550552 | atg16l1 |  |  | 13427 | 0.119 | -0.1471 | No |
| 29 | 393210 | wdr45b |  |  | 13916 | 0.096 | -0.1606 | No |
| 30 | 492805 | nsfl1c |  |  | 14202 | 0.082 | -0.1680 | No |
| 31 | 100009663 | atg9a |  |  | 14551 | 0.067 | -0.1776 | No |
| 32 | 494180 | atg5 |  |  | 14638 | 0.063 | -0.1791 | No |
| 33 | 100003524 | retreg1 |  |  | 14719 | 0.059 | -0.1805 | No |
| 34 | 565165 | si:ch1073-143l10.2 |  |  | 14905 | 0.051 | -0.1854 | No |
| 35 | 100034627 | atg16l2 |  |  | 14974 | 0.048 | -0.1866 | No |
| 36 | 563215 | vps41 |  |  | 15244 | 0.035 | -0.1944 | No |
| 37 | 403036 | gabarapl2 |  |  | 15513 | 0.022 | -0.2024 | No |
| 38 | 550597 | ei24 |  |  | 15575 | 0.018 | -0.2040 | No |
| 39 | 564072 | tp53inp2 |  |  | 15680 | 0.013 | -0.2070 | No |
| 40 | 436933 | sesn1 |  |  | 16367 | -0.013 | -0.2283 | No |
| 41 | 556500 | tecpr1a |  |  | 16379 | -0.013 | -0.2284 | No |
| 42 | 558848 | ulk1b |  |  | 17219 | -0.042 | -0.2540 | No |
| 43 | 337168 | nbr1a |  |  | 17247 | -0.043 | -0.2540 | No |
| 44 | 100124616 | zgc:171927 |  |  | 17396 | -0.050 | -0.2577 | No |
| 45 | 564088 | pik3r4 |  |  | 17575 | -0.057 | -0.2623 | No |
| 46 | 541415 | zgc:101577 |  |  | 17999 | -0.075 | -0.2741 | No |
| 47 | 560881 | mtm1 |  |  | 18231 | -0.087 | -0.2797 | No |
| 48 | 556338 | tbc1d25 |  |  | 18255 | -0.088 | -0.2788 | No |
| 49 | 406840 | sesn3 |  |  | 18281 | -0.089 | -0.2779 | No |
| 50 | 324307 | atp6v0a1a |  |  | 18391 | -0.093 | -0.2795 | No |
| 51 | 393184 | clec16a |  |  | 18413 | -0.094 | -0.2784 | No |
| 52 | 565649 | fyco1a |  |  | 18448 | -0.096 | -0.2776 | No |
| 53 | 101883461 | si:ch73-45o6.2 |  |  | 18697 | -0.108 | -0.2833 | No |
| 54 | 563679 | zgc:136908 |  |  | 18750 | -0.111 | -0.2829 | No |
| 55 | 100000896 | rubcn |  |  | 18805 | -0.114 | -0.2824 | No |
| 56 | 393362 | wipi1 |  |  | 19013 | -0.122 | -0.2866 | No |
| 57 | 561574 | si:ch211-260b17.6 |  |  | 19278 | -0.134 | -0.2923 | No |
| 58 | 566014 | retreg3 |  |  | 19447 | -0.142 | -0.2949 | No |
| 59 | 100151338 | tecpr1b |  |  | 19485 | -0.143 | -0.2933 | No |
| 60 | 407723 | smcr8b |  |  | 19750 | -0.155 | -0.2986 | No |
| 61 | 567680 | atg101 |  |  | 19792 | -0.157 | -0.2969 | No |
| 62 | 437002 | wipi2 |  |  | 20125 | -0.172 | -0.3041 | No |
| 63 | 407686 | zgc:113944 |  |  | 20499 | -0.188 | -0.3122 | No |
| 64 | 323208 | tmem39a |  |  | 20698 | -0.199 | -0.3147 | No |
| 65 | 564989 | nprl3 |  |  | 20758 | -0.202 | -0.3127 | No |
| 66 | 393200 | wdr45 |  |  | 21298 | -0.230 | -0.3252 | No |
| 67 | 100332088 | tbc1d12b |  |  | 22050 | -0.270 | -0.3437 | No |
| 68 | 100333062 | wdr81 |  |  | 22198 | -0.278 | -0.3430 | No |
| 69 | 562638 | scoca |  |  | 22330 | -0.286 | -0.3416 | No |
| 70 | 393405 | atg13 |  |  | 22362 | -0.287 | -0.3371 | No |
| 71 | 550411 | yod1 |  |  | 22366 | -0.288 | -0.3317 | No |
| 72 | 406452 | sqstm1 |  |  | 22703 | -0.308 | -0.3364 | No |
| 73 | 449761 | si:ch211-163l21.8 |  |  | 22839 | -0.316 | -0.3346 | No |
| 74 | 337424 | arl8ba |  |  | 23120 | -0.335 | -0.3370 | No |
| 75 | 393846 | becn1 |  |  | 23266 | -0.345 | -0.3349 | No |
| 76 | 322756 | ufm1 |  |  | 23624 | -0.369 | -0.3391 | No |
| 77 | 548346 | pik3c3 |  |  | 24150 | -0.403 | -0.3479 | No |
| 78 | 100149048 | si:zfos-80g12.1 |  |  | 24470 | -0.418 | -0.3500 | No |
| 79 | 100333016 | atg7 |  |  | 25106 | -0.454 | -0.3612 | No |
| 80 | 100149745 | sesn2 |  |  | 25398 | -0.475 | -0.3613 | No |
| 81 | 641330 | atg10 |  |  | 25462 | -0.481 | -0.3541 | No |
| 82 | 402960 | tex264a |  |  | 26566 | -0.584 | -0.3776 | Yes |
| 83 | 386712 | uba5 |  |  | 26580 | -0.586 | -0.3668 | Yes |
| 84 | 567385 | vti1a |  |  | 26783 | -0.607 | -0.3616 | Yes |
| 85 | 368883 | rab1ab |  |  | 26896 | -0.619 | -0.3533 | Yes |
| 86 | 393268 | map1lc3c |  |  | 27020 | -0.631 | -0.3451 | Yes |
| 87 | 791709 | scocb |  |  | 27485 | -0.671 | -0.3468 | Yes |
| 88 | 326974 | gabarapa |  |  | 27990 | -0.740 | -0.3485 | Yes |
| 89 | 322425 | map1lc3b |  |  | 28058 | -0.750 | -0.3363 | Yes |
| 90 | 559366 | lrrk2 |  |  | 28112 | -0.758 | -0.3234 | Yes |
| 91 | 561208 | tmem41b |  |  | 28148 | -0.764 | -0.3099 | Yes |
| 92 | 394011 | vti1b |  |  | 28249 | -0.778 | -0.2982 | Yes |
| 93 | 553778 | rab23 |  |  | 28401 | -0.804 | -0.2876 | Yes |
| 94 | 563626 | tbc1d12a |  |  | 28470 | -0.814 | -0.2741 | Yes |
| 95 | 557744 | tbc1d14 |  |  | 28591 | -0.838 | -0.2619 | Yes |
| 96 | 572161 | lix1l |  |  | 28784 | -0.875 | -0.2512 | Yes |
| 97 | 100037316 | ulk3 |  |  | 28900 | -0.899 | -0.2376 | Yes |
| 98 | 393263 | ddrgk1 |  |  | 29694 | -1.126 | -0.2410 | Yes |
| 99 | 327253 | hdac10 |  |  | 30271 | -1.377 | -0.2328 | Yes |
| 100 | 569543 | epg5 |  |  | 31388 | -2.622 | -0.2177 | Yes |
| 101 | 799873 | spns1 |  |  | 31576 | -3.178 | -0.1628 | Yes |
| 102 | 327197 | vcp |  |  | 31772 | -4.315 | -0.0864 | Yes |
| 103 | 100000242 | zgc:92606 |  |  | 31809 | -4.711 | 0.0025 | Yes |
Table: GSEA details
[plain text format]

  

Fig 2: MACROAUTOPHAGY(GO:0016236)
  
Blue-Pink O' Gram in the Space of the Analyzed GeneSet

  

Fig 3: MACROAUTOPHAGY(GO:0016236): Random ES distribution
  
Gene set null distribution of ES for
 **MACROAUTOPHAGY(GO:0016236)**
